# Predominant *Campylobacter jejuni* Sequence Types Persist in Finnish Chicken Production

**DOI:** 10.1371/journal.pone.0116585

**Published:** 2015-02-20

**Authors:** Ann-Katrin Llarena, Adeline Huneau, Marjaana Hakkinen, Marja-Liisa Hänninen

**Affiliations:** 1 Department of Food Hygiene and Environmental Health, Faculty of Veterinary Medicine, University of Helsinki, Helsinki, Finland; 2 Anses, Ploufragan-Plouzané laboratory, BP 53, 22440, Ploufragan, France; 3 Food and Feed Microbiology Research Unit, Research and Laboratory Department, Finnish Food Safety Authority, Evira, Helsinki, Finland; St. Petersburg Pasteur Institute, RUSSIAN FEDERATION

## Abstract

Consumption and handling of chicken meat are well-known risk factors for acquiring campylobacteriosis. This study aimed to describe the *Campylobacter jejuni* population in Finnish chickens and to investigate the distribution of *C. jejuni* genotypes on Finnish chicken farms over a period of several years. We included 89.8% of the total *C. jejuni* population recovered in Finnish poultry during 2004, 2006, 2007, 2008, and 2012 and used multilocus sequence typing (MLST) and pulsed-field gel electrophoresis (PFGE) to characterize the 380 isolates. The typing data was combined with isolate information on collection-time and farm of origin. The *C. jejuni* prevalence in chicken slaughter batches was low (mean 3.0%, CI_95%_ [1.8%, 4.2%]), and approximately a quarter of Finnish chicken farms delivered at least one positive chicken batch yearly. In general, the *C. jejuni* population was diverse as represented by a total of 63 sequence types (ST), but certain predominant MLST lineages were identified. ST-45 clonal complex (CC) accounted for 53% of the isolates while ST-21 CC and ST-677 CC covered 11% and 9% of the isolates, respectively. Less than half of the *Campylobacter* positive farms (40.3%) delivered *C. jejuni*-contaminated batches in multiple years, but the genotypes (ST and PFGE types) generally varied from year to year. Therefore, no evidence for a persistent *C. jejuni* source for the colonization of Finnish chickens emerged. Finnish chicken farms are infrequently contaminated with *C. jejuni* compared to other European Union (EU) countries, making Finland a valuable model for further epidemiological studies of the *C. jejuni* in poultry flocks.

## Introduction

*Campylobacter* spp. is the most common causative agent for bacterial gastroenteritis worldwide, including Finland [1,2]. In 1999, campylobacteriosis surpassed the number of salmonellosis cases, and has since been the nation’s most important bacterial zoonosis with 4064 registered Finnish cases in 2013 [3,4]. The majority of cases (95%) are caused by *Campylobacter jejuni*, which upon ingestion normally results in a self-limiting gastroenteritis, and the number of cases peaks during the summer months of July and August [5].

*C*. *jejuni* have been isolated from a wide range of animal species and environmental sources [6–8]. Several studies have shown that eating and handling of improperly cooked or raw poultry meat poses a risk for contracting campylobacteriosis [9–11]. Between 2004 and 2013, both the Finnish production and consumption of broiler meat has increased (http://www.siipi.net/index.php/siipikarjaliitto/tilastoa) [12]. The number of rearing farms has stayed stable, at approximately 140 during this period (epp.eurostat.ec.europa.eu), while the average farm and flock sizes have increased to approximately 35 000 birds per farm raised in houses accommodating 15–20 000 animals (www.mmm.fi). The farms work on a contractual basis with one of three Finnish chicken slaughterhouses, and depopulation strategies, like thinning, are not practiced. Instead, the all-in all-out system with an empty period from one to four weeks is utilized [13].

EU-member states are required to monitor the occurrence of *Campylobacter* in the chicken chain, which in Finland is accomplished through the compulsory *Campylobacter* monitoring program for chicken slaughter batches [14]. The prevalence of *Campylobacter*-positive slaughter batches in different EU-member states varies markedly, with big chicken-producing countries like the United Kingdom and Spain having a prevalence ranging from 59.6% to 82.2%, whereas the corresponding figures in the Nordic countries of Norway, Sweden, and Finland have been considerably lower. For the period 2004–2012, the annual *C*. *jejuni* prevalence in Finnish chicken batches has ranged between 2.1% and 7.0% [15,16].

The European Food Safety Authority (EFSA) Panel on Biological Hazards (BIOHAZ) estimated that 50–80% of all campylobacteriosis cases are associated with the chicken reservoir as a whole. The panel has indicated that a reduction in the number of *Campylobacter* spp. positive flocks would be the most cost-effective measure in controlling human campylobacteriosis [17,18]. To reduce the occurrence of *C*. *jejuni* in European chicken flocks, information on possible contamination pathways to the flocks is needed. Due to this bacterium’s wide host range, high diversity, and weakly clonal population, subtyping of *C*. *jejuni* below the species level is necessary to achieve effective source tracking [19]. Genotyping methods such as pulsed-field gel electrophoresis (PFGE) and multilocus sequence typing (MLST) have been used to characterize the *C*. *jejuni* and *C*. *coli* populations in chickens during the last decade, and recently next-generation sequencing has been increasingly utilized to generate high-resolution full-genome data in molecular epidemiology [20]. While PFGE has a high discriminatory power and has been used successfully in focused short-term epidemiological studies, MLST is especially well adapted to long-term epidemiological investigation and source attribution [21]. Several studies have found shared PFGE and MLST types between *C*. *jejuni* isolated from human patients and chickens, thereby emphasizing the important role this reservoir plays in the epidemiology of campylobacteriosis [7,22,23]. In addition, PFGE and MLST have been used to investigate possible contamination sources on isolated chicken farms [24]. When similar genotypes of *C*. *jejuni* from the environment surrounding a chicken house or certain animal hosts and chickens are found, these vehicles and reservoirs may be acting as a contamination source of the chickens [24,25]. However, studies attempting to link chicken-derived *C*. *jejuni* to a larger number of farms over a longer time-span are lacking, even though such characterization data could provide valuable information on the nature of *C*. *jejuni* colonization in this reservoir, benefitting development of on-farm intervention strategies.

This study investigated *C*. *jejuni* populations by MLST and PFGE among Finnish chicken slaughter batches sampled over a nine-year period through the official monitoring program and identified possible dynamic effects of year, season, and area of slaughter on this *C*. *jejuni* population. The resulting MLST and PFGE data was further linked to information about farm origin to investigate the possibility of a persistent colonization source on certain farms.

## Materials and Methods

### *C*. *jejuni* isolates

This study included *C*. *jejuni* isolates collected in the Finnish *Campylobacter* monitoring program for poultry during 2004, 2006–2008 and 2012 [14]. According to this surveillance program, all chicken batches slaughtered between June and October undergo compulsory testing for the presence of *C*. *jejuni* and *C*. *coli*. The sampling from January to May and in November and December was randomized using an expected target prevalence of 5% (1% since 2008), confidence level of 95%, and desired precision of 5% (1% since 2008). In 2004, no sampling took place between January and May, but the described randomized sampling above was utilized between November and December. During the five-year study period, a total of 7894 chicken batches were tested; 7070 were slaughtered between June and October, and 824 were slaughtered between October and June.

The detection of *C*. *jejuni* was done according to the method of Finnish Food Safety Authority (Evira) number 3512/5 [26]. Sampling was conducted at the time of slaughter at all three Finnish slaughterhouses handling poultry, and one sample consisted of 10 intact ceca per slaughter batch. The cecal contents were then pooled into a 5-ml sterile water suspension. A 10-μl loop-full of this suspension was cultured onto a ***Campylobacter* blood-free selective agar** (mCCDA) (media supplier varies between different slaughter-house laboratories) and incubated under microaerobic conditions (5% O_2_, 10% CO_2_, 85% N_2_) at 41.5 ± 0.5°C for 24–48 hours. In the event of typical *Campylobacter* growth on the mCCDA plates, one colony was sub-cultivated and sent to Evira for confirmation tests (ISO 10272–1:2006). The isolates were stored in Brucella broth (Cat. no. 211088, BD Biosciences, Vantaa, Finland) supplemented with 15% glycerol at -70°C to await further analysis.

During the five study years, a total of 423 chicken batches were positive for *C*. *jejuni*, and the distribution of the isolates between study-years was 82, 66, 95, 98 and 82 for 2004, 2006, 2007, 2008, and 2012, respectively. Almost all isolates were collected between June and October. The overall mean *C*. *jejuni* prevalence was 3.0%, CI_95%_ [1.8%, 4.2%], with a range of 2.1% to 4.5%. Furthermore, the overall prevalence for *C*. *jejuni* during June to October was 5.8%, CI_95%_ [4.9%, 6.7%] [27,28] (Table 1).

**Table 1:** Overview of *Campylobacter jejuni* prevalence in Finnish chicken slaughter batches, the most common multilocus sequence types (STs) found among these isolates in 2004, 2006, 2007, 2008, and 2012 and farm-related data according to year of study and number of positive *C*. *jejuni* batches per farm.

	Year					
	2004	2006	2007	2008	2012	All years
Prevalence Summer	5.6%	5.0%	6.6%	6.4%	5.3%	5.7% CI_95%_ [4.9%, 6.7%]
Prevalence Winter	0.0%_A_	0.0%	0.0%	3.2%	1.6%	0.9% CI_95%_[0%, 2.7%]
Pos. batches (total)_B_	69/ 50*	60/ 47*	88/ 59*	78/ 58*	85/ 58*	380/ 273*
Common CCs _C_	ST-45 CC (58.8%)	ST-45 CC (51.1%)	ST-45 CC (42.4%)	ST-45 CC (51.7%)	ST-45 CC (65.5%)	ST-45 CC (53.8%)
	ST-677 CC (9.8%)	ST-283 CC (14.9%)	ST-677 CC (15.3%)	ST-21 CC (20.7%)	ST-677 CC (10.3%)	ST-677 CC (10.6%)
	ST-21 CC (9.8%)	ST-677 CC, ST-21 CC (8.5% both)	ST-21 CC (6.8%)	ST-677 CC (8.6%)	ST-283 CC (8.6%)	ST-21 CC (9.2%)
Common STs _C_	ST-45 (35.3%)	ST-45 (38.3%)	ST-45 (18.6%)	ST-45 (27.6%)	ST-45 (46.6%)	ST-45 (33.0%)
	ST-230 (13.7%)	ST-267 (14.9%)	ST-677 (13.6%)	ST-451 (15.5%)	ST-11 (12.1%)	ST-677 (9.5%)
	ST-677 (9.8%)	ST-677 (8.5%)	ST-3805 (6.8%)	ST-677 (8.6%)	ST-267 (8.6%)	ST-267, ST-230 (5.5% both)
Common PFGE_B_	K36 (12.2%)	K36, S54 (10.6% both)	S110 (9.8%)	S12 (20.4%)	S54 (16.1%)	K36 (7.8%)
	S4, S64 (8.2% both)	S64 (8.5%)	S64 (9.6%)	S7 (9.3%)	S4 (14.3%)	S54 (7.4%)
	S7 (6.1%)	S66 (6.4%)	S55 (7.7%)	S5 (7.4%)	S7 (8.9%)	S64 (7.0%)
Farms						
Delivering to slaughter	n = 143	n = 124	n = 138	n = 141	n = 124	Mean n = 135.7 CI_95%_ [125.9,145.4]
C. jejuni positive	n = 32 (22.4%)	n = 30 (25.8%)	n = 45 (32.6%)	n = 39 (27.7%)	n = 40 (32.2%)	114 (84.0%)

_A_ Winter prevalence in 2004 was calculated on sampling from November and December only

_B_ Absolute numbers of positive chicken batches according to year both before and after adjusting for clustering of flocks within farms (adjusted database marked with asterisk).

_C_ The three most common CCs, STs and PFGEs reported with their respective percentages of isolates according to year of collection. All percentages are calculated on the basis of the adjusted database: isolates originating from the same farm and week with identical MLST and PFGE types counts as one isolate to correct for clustering of flocks within the same farm.

CC: Clonal complex

PFGE: Pulsed-Field Gel Electrophoresis

### MLST typing

The *C. jejuni* isolates (n = 380) included comprised 89.8% of all isolates acquired from the monitoring program in 2004, 2006, 2007, 2008 and 2012. MLST typing for 147 isolates from 2006 and 2007 was performed in an earlier study [22]. Of the remaining 233 *C. jejuni* isolates, the sequence type (ST) of 48 isolates was determined by the use of a published *C. jejuni* MLST protocol [29,30]. For 185 isolates, the MLST types were determined using Illumina next generation genome sequencing technology as described elsewhere [31].

### PFGE typing

To achieve a higher discriminatory power in the analysis of the *C*. *jejuni* population on each farm, a total of 366 *C*. *jejuni* isolates was subtyped by pulsed-field gel electrophoresis (PFGE) with SmaI as a restriction enzyme as described by Hakkinen et al. (2007). Isolates for which the DNA was not digestable by SmaI were subtyped using KpnI for restriction as described by Hakkinen et al. (2009) [32,33]. Subtypes obtained by SmaI and KpnI restriction were named S1, S2 etc., and K1, K2 etc., respectively.

### Creation of the adjusted database

To account for potential *C*. *jejuni* genotype clusters within the flocks on the chicken farms, isolates having a similar MLST and PFGE profiles from batches originating from the same farm and collected within a one-week interval (one rearing cycle) were merged to account for one isolate. In cases where no PFGE profile was available and two isolates were of the same ST (n = 4), both isolates were included in the adjusted database. This database was used in the descriptive analysis of the MLST and PFGE genotypes, in the analysis of the association between MLST and PFGE genotypes and in the logistic regression analysis, while the full database was used in the farm-associated data description.

### Statistics

All data handling and statistics were done in IBM SPSS version 21 (International Business Machines Corp., Armonk, NY, USA), except for the logistic regression analysis which was carried out in SAS version 9.4 (SAS Institute Inc., Cary, NC, USA). Diversity calculations were done in PAST version 3.01 [34]. Calculation of a potential association between CC and ST with PFGE SmaI types was done using Fisher’s exact test.

Logistic regression analysis was employed to predict the probability that an isolate belonged to one of the three most common CCs (ST-45 CC, ST-677 CC, ST-21 CC) or the most common ST (ST-45). For each CC or ST, the model was developed to include year of collection (variable Year; categories “2004”, “2006”, “2007”, “2008” and “2012”) and area of slaughter (variable Area; categories “A”, “B” and “C”) as predictive variables. For ST-45 CC and ST-45, sufficient number of isolates was achieved to include the effect of season, and logistic models were built up including the predictive variables of Area, Year, and Period. The variable Period consisted of concatenated months of collection (variable Period; categories “January to June”, “July”, “August”, “September”, and “October to December”) reflecting biologically sensible seasons and relevant high peak months to achieve a sufficient number of isolates in all categories. The categories of reference were chosen to simplify the interpretation of the computed Odds Ratios (ORs). Due to observed over-dispersion, the model was fit using a scale parameter estimated under the full model (procedure LOGISTIC, scaled using William’s method) and goodness of fit was tested by the Hosmer and Lemeshow method. Interaction between variables was not assessed due to the low number of observations. Only results from regression analysis in which the test of the full model versus the model with an intercept was statistically significant (p < 0.05) are reported.

## Results

### MLST and PFGE typing of the *C*. *jejuni* population

From the 380 isolates, 378 isolates were successfully MLST typed (69, 60, 88, 78 and 83 from 2004, 2006, 2007, 2008 and 2012, respectively) and assigned into a total of 63 STs, of which eight were novel (ST-3865, ST-6236, ST-6237, ST-6555, ST-6571, ST-7008, ST-7011, ST-7020). Of these 63 STs, 34 clustered into 12 CCs while a substantial proportion of the STs (n = 28, 44.4%), containing 44 isolates (11.6%), were unassigned to any clonal complex (analysis time July 2014) (S1 Table). Of the 380 isolates, 273 strains were included in the adjusted database (51, 47, 59, 58 and 58 from 2004, 2006, 2007, 2008 and 2012, respectively). Simpson’s diversity index was 0.87, CI_95%_ [0.86, 0.91], based on ST distribution. The most common CCs were ST-45 CC (53.8%), ST-677 CC (10.6%), ST-21 CC (9.2%), and ST-283 CC (5.5%). Thirty-six isolates (13.2%) were not members of any clonal complex (Table 1 and S1 Table) and the remaining CCs (n = 9) each represented less than 2% of the isolates. The most common STs were ST-45 (33.0%), ST-677 (9.5%), ST-267 (5.5%), ST-230 (5.5%), ST-451 (4.8%), ST-11 (4.4%), ST-50 (2.9%), ST-1326 (2.2%), and ST-1367 (2.2%) (Table 1 and S1 Table).

In the adjusted database, a total of 226 and 32 isolates were successfully PFGE typed with SmaI and KpnI, respectively. K36 (7.8%), S54 (7.4%), S64 (7.0%), S7 (6.2%), S4 (6.2%), S66 (4.7%), S12 (4.3%), S55 (3.9%), S78 (3.1%), and S74 (3.1%) were the most frequently isolated PFGE genotypes. The remaining PFGE SmaI and KpnI profiles accounted for less than 3% of the isolates.

The three most common CCs, ST-45 CC, ST-677 CC and ST-21 CC, gave rise to 40, 9, and 17 PFGE types, respectively. Certain PFGE types associated significantly with the ST-45 CC; S4 (p<0.0001), S54 (p = 0.005), S55 (p = 0.002), S66 (p = 0.04), and S74 (p = 0.008). ST-677 CC was associated with PFGE SmaI types S64 (p<0.0001) and S78 (p<0.0001). ST-21 CC was equally distributed among the PFGE types, and did therefore not associate with any specific PFGE type.

ST-45 was found to be the most diverse ST with 23 different PFGE types. Of these, S4 (p<0.0001), S55 (p = 0.001), S66 (p = 0.0006), and S7 (p = 0.0008) were associated with this ST. ST-677, accounting for a total of 10 PFGE genotypes, was significantly correlated with the PFGE types S64 (p<0.0001) and S78 (p<0.0001), while ST-11 and ST-267 were associated with the presence of S54 (p<0.0001) and K36 (p<0.0001), respectively.

### Association with farm data

**Fig 1:**
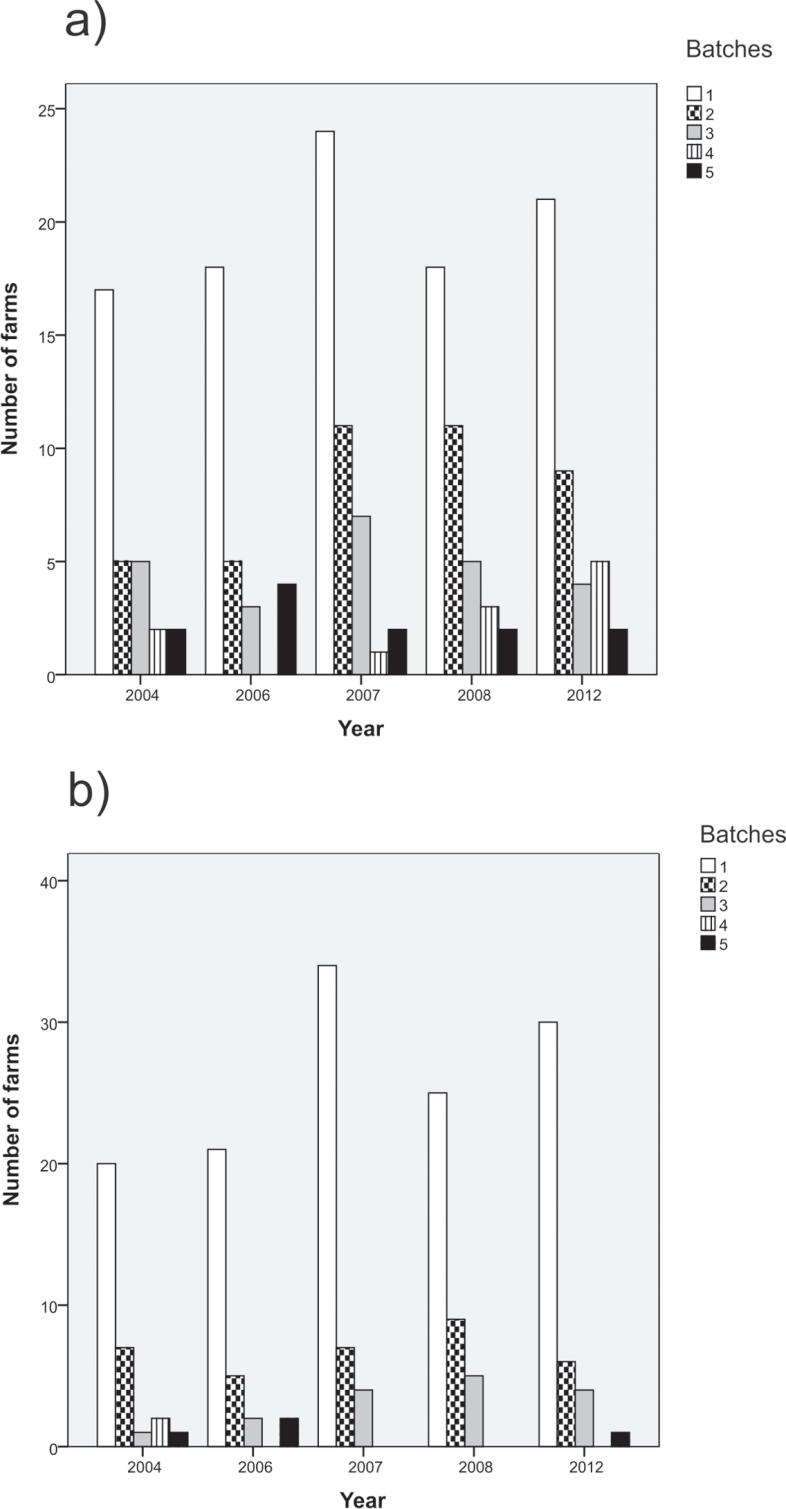
Number of farms during the five study years grouped by the frequency of *C*. *jejuni* positive chicken batches delivered to slaughter annually (data on farms which delivered no positive batches has been omitted). In Fig. 1A) the numbers are counted on the basis of all 380 *C*. *jejuni* isolates acquired in the study, whereas in Fig. 1B) the numbers are based on the adjusted database. In the latter database, isolates with similar MLST and PFGE types collected within a week of each other and originating from the same farm was counted as one clone to account for clustering of flocks within the farms.

Over the study period, an annual mean of 135.7 (CI_95%_ [125.9, 145.4]) farms reared and delivered chicken to slaughter, with an yearly average of 37.0 (CI_95%_ [29.8, 44.2]) farms having *C*. *jejuni*-positive chicken batches. The 380 *C*. *jejuni* isolates included in the study represented 114 of the 118 farms delivering *C*. *jejuni*-positive chicken batches during the five study years (96.6%). Most farms delivered only one positive batch annually (Fig. 1) or delivered *C*. *jejuni*-positive batches in only one of the five study years. More specifically, 68 (59.6%), 25 (21.9%), 17 (14.9%), and 4 (3.5%) of the 114 farms delivered at least one *C*. *jejuni*-positive batch in one, two, three, or four of the five study years, respectively. The distribution of *C*. *jejuni*-positive farms according to study year is given in Table 1.

Of the farms delivering *C*. *jejuni*-positive batches over multiple years (n = 46), the same ST was found on 14 farms (30.4%), but in only two of these cases were the isolates had a similar PFGE genotype. On Farm B10, ST-45/SmaI S7 was isolated both in both 2004 and 2007 and on Farm C14 ST-45/SmaI S66 was isolated in both 2006 and 2007. On the other hand, within each year nearly half of the *C*. *jejuni* positive farms (48.2%) delivered two or more chicken batches during the same rearing cycle (i.e. within one week) as these chickens originated from different houses on the same farm. From the majority of these farms (76.3%), two or more isolates of the same PFGE type were identified.

Among the study farms, ten were “high-frequency” farms, producing 4% (Farm B1), 3% (Farms B5, C5), and 2% (Farms A11, A12, A19, C4, C8, C28, C38) of the *Campylobacter*-positive batches. The *C*. *jejuni* isolates originating from these ten farms had a total of 29 STs assigned to 8 CCs, while 11 STs and 12 isolates remained unassigned to any clonal complexes. Isolates collected from three farms (Farms B1, C4, and C5) were found to have the same STs persisting over two consecutive study years or longer, however, the PFGE profiles of the isolates varied (Table 2, marked with an asterisk). No persistent ST over several study years was found in batches originating from the remaining seven “high-frequency” farms. Furthermore, isolates from batches representing different rearing cycles within one year had different MLST and PFGE profiles on the majority of these farms (Table 2).

**Table 2:** Distribution of multilocus sequence types on the 10 farms representing over 2% of the 378 MLST-typeable isolates during the five study years according to month.

Time-period		Farms									
Year	Month	A11	A12	A19	B1	B5	C4	C5	C8	C28	C38
2004	June				ST-50/ S90_S_						
					ST-45*/ S66_S_						
	July				ST-45*/ S7			ST-451/ S5	ST-230/ K36	ST-230/ nd	
	Aug				ST-677/ S8	ST-1539/ S96_S_				ST-230/ K59_S_	
						ST-3477/ K60_S_				ST-677/ S64_S_	
	Sep								ST-45/ S54_S_		
									ST-230/ K36_S_		
	Oct					ST-53/ S1_S_					
						ST-4596/ K78_S_					
	Nov					ST-4596/ K78					
										
2006	June		ST-45/ S64				ST-3999/ K73	ST-267/ S54_S_			
								ST-583/ S64_S_			
	July				ST-267/ S64_S_						
					ST-267/ S102_S_						
					ST-267/ K36_S_						
					ST-45*/ S14_S_						
	Aug				ST-267/ K36			ST-3453/ S162_S_			
								ST-50/ S101_S_			
	Sep			ST-45/ S74_S_				ST-586/ S13			ST-45/ S55
				ST-45/ S54_S_							
				ST-45/ S4_S_							
	Oct	ST-4000/ S155									
2007	June		ST-3805 / S110								
	July	ST-3805/ S110			ST-45*/ S55		ST-993 / S117				
	Aug			ST-356/ S113_S_		ST-45/ S55_S_		ST-1326*/ S120			
				ST-356/ S103_S_		ST-677/ S64_S_					
	Sep	ST-1326/ S14					ST-677*/ S64_S_				
							ST-1278/ S122_S_				
	Oct		ST-4003/ S108			ST-45/ S55					
2008	June						ST-50/ S130	ST-50/ S130			
	July		ST-6228/ S161_S_			ST-451/ S12		ST-1326*/ S14			
			ST-1080/ S7_S_								
	Aug		ST-692 / nd		ST-451/ S12		ST-677*/ S86_S_		ST-230/ S106_S_	ST-45/ S74_S_	ST-2219/ S78_S_
					ST-451/ S12_S_		ST-137/ S157_S_		ST-1326/ S158_S_	ST-137/ K61_S_	ST-677/ S78_S_
					ST-451/ 171_S_						
	Sep	ST-45/ S120_S_									
		ST-692/ S120_S_									
	Oct			ST-45/ S7							
	Nov	ST-4307/ S156									
2012	March										ST-45/S150
	May										ST-1276/nd
	July								ST-230/ S14_S_		ST-267/ K36
									ST-45/ S4_S_		ST-267/ K36
	Aug	ST-11/ S54									
	Sep									ST-267/ K36	
	Oct										ST-45/S150

The table is based on the adjusted database: isolates originating from the same farm and week with identical MLST and PFGE types counts as one isolate to correct for clustering of flocks within the same farm. Empty cells represent time-periods when no *C*. *jejuni* positive chicken batches were registered from the respective farms.

*: Same ST over multiple years

_S_: isolates collected during the same rearing cycle

nd: PFGE not done

ST: Sequence type

S: SmaI type

K: KpnI type

### Spatial and temporal variation in genotypes

For the isolates included in the study, a clear seasonal trend in the occurrence of *Campylobacter* was observed, with 70.6% of the isolates collected in July and August (S2 Table).

Logistic regression analysis was carried out to investigate the effect of year of collection, site of slaughter, and, for ST-45 CC and ST-45, season on the presence or absence of the most common CCs and STs among the chicken-derived *C*. *jejuni* population. The results of the goodness of fit test (Hosmer and Lemeshow) are presented in Table 3 under their respective model.

**Table 3:** Results of multiple logistic regression analysis used to predict the probability that a *C*. *jejuni* isolate belongs to ST-45 CC, ST-21 CC, ST-677 CC, or ST-45.

		CC			ST
Model parameters		ST-45 CC	ST-21 CC	ST-677 CC	ST-45
	Overall model_A_	P = 0.04	P = 0.002	P = 0.84	P = 0.003
	Goodness of fit_B_	P = 0.46	P = 0.53	P = 0.69	P = 0.12
Predictor variable					
Year	P	0.15	0.03*	0.86	0.12
		OR w IC_95%_	OR w IC_95%_	OR w IC_95%_	OR w IC_95%_
	2004	1	1	1	1
	2006	0.65 [0.28, 1.50]	1.30 [0.31, 5.42]	0.87 [0.21, 3.52]	1.20 [0.48, 2.99]
	2007	0.44 [0.19, 1.03]	1.12 [0.27, 4.75]	1.53 [0.45, 5.25]	0.46 [0.17, 1.21]
	2008	0.67 [0.29, 1.46]	4.58* [1.37, 15.30]	0.86 [0.23, 3.29]	0.71 [0.29, 1.76]
	2012	1.14 [0.50, 2.61]	Nd	1.07 [0.30, 3.86]	1.91 [0.80, 4.57]
Area	P	0.03*	0.001*	0.61	0.31
		OR w IC_95%_	OR w IC_95%_	OR w IC_95%_	OR w IC_95%_
	A	2.74* [1.30, 5.76]	1	1	0.68 [0.30, 1.53]
	B	1	13.5* [3.30, 58.20]	1.28 [0.46, 3.54]	1
	C	2.17* [1.03, 4.32]	4.77* [1.23, 18.50]	0.74 [0.29, 1.91]	0.54 [0.25, 1.19]
Period	P	0.27	nd	nd	<0.001*
		OR w IC_95%_	OR w IC_95%_	OR w IC_95%_	OR w IC_95%_
	Jan-June	1			1
	July	2.22 [0.75, 6.55]			2.18 [0.64, 7.39]
	August	1.24 [0.41, 3.68]			0.58 [0.15, 2.14]
	September	2.01 [0.61, 6.60]			4.09* [1.09, 15.30]
	Oct-Dec	1.81 [0.48, 6.81]			5.29* [1.24, 22.60]

The predictor variables were year of collection (Year), period of collection (Period), and site of slaughter (Area). The odds ratio with 95% Wald confidence limits are listed for each of the ORs. Reference categories are depicted with OR = 1.

* Significant OR

_A_ Test of the full model versus a model with only intercept (Wald test)

_B_ Hosmer and Lemeshow test

CC: Clonal complex

ST: Sequence Type

nd: Not done

The ST-45 CC was the most prevalent complex during all study years. The odds of a *C*. *jejuni* isolate being of this clonal complex were higher in Area A (OR 2.74, IC_95%_ [1.30, 5.76]) and Area C (OR 2.17, IC_95%_ [1.03, 4.32]) than in Area B. No influence of the month of sampling or year of collection on the frequency of ST-45 CC was observed.

As no strain belonging to the ST-21 CC complex was isolated in 2012, the logistic regression model was constructed excluding this sampling year. Both area of slaughter and year of collection had a significant effect on the occurrence of ST-21 CC among *C*. *jejuni*-positive chicken flocks. An isolate from 2008 was more likely to be of the ST-21 CC than an isolate from 2004 (OR: 4.58, IC_95%_ [1.37–15.30]). Furthermore, the ST-21 CC appeared to be more frequently isolated from the flocks slaughtered in Areas B (OR: 13.5, IC_95%_ [3.30, 58.20]) and C (OR: 4.77, IC_95%_ [1.23, 18.50]) than in Area A.

The odds for a *C*. *jejuni*-positive chicken flock to be of the ST-677 CC were equally distributed among years of collection and site of slaughter such that no effect of either of these predictor variables could be seen.

ST-45 was the most common ST isolated from our study and represented 61.2% of the isolates belonging to the ST-45 CC complex. This ST was more commonly isolated in September (OR: 4.09, IC_95%_ [1.09, 15.30]) and from October to December (OR: 5.29, IC_95%_ [1.24, 22.60]) than from January to June. The area of slaughter or year of collection had no influence on the probability of detecting an ST-45 strain in a positive chicken flocks.

## Discussion

Our study included an average of 136 chicken farms each year and encompassed a total of 7894 chicken batches representing estimated 276 million birds slaughtered during five study years. Finland has a low prevalence of *C*. *jejuni* in chicken slaughter batches relative to the occurrence in other EU member states [28], making Finland a valuable model for studying the epidemiology of *Campylobacter* distribution in poultry flocks. We found that each year approximately a quarter of Finnish chicken farms delivered *C*. *jejuni*-colonized batches to slaughter and over half (59.6%) of the 114 farms from which *C*. *jejuni*-positive batches were obtained delivered positive batches in only one of the study years. Even though the *C*. *jejuni* population in Finnish chickens was diverse as assessed by MLST and PFGE typing, the composition of it was relatively stable within the study-period due to the predominance of certain MLST types.

The inclusion of 89.8% of all *C*. *jejuni* isolates acquired from the Finnish *Campylobacter* monitoring program for chickens during the study period resulted in a representative collection of the *C*. *jejuni* population of this reservoir. According to the sampling scheme, 10 bird ceca from each batch are pooled and analyzed for the presence of *C*. *jejuni* and *C*. *coli*. The inclusion of one isolate from each *C*. *jejuni*-positive chicken batch was deemed adequate for estimating the *Campylobacter* status of the chicken flock as Finnish studies have previously shown that once *Campylobacter* colonization is detected, the majority of the birds in the same flock are colonized by a single PFGE type (Hakkinen and Kaukonen, 2009, presented at the 15^th^ International Workshop on *Campylobacter*, *Helicobacter* and Related Organisms, Niigata, Japan, 2–9. September). Our results are in line with this finding since we found that *C*. *jejuni* isolates collected from different batches during one rearing cycle were of the same PFGE type on the majority of farms (76.3%). Nevertheless, the possibility that some minor genotypes were missed due to the sampling design cannot be ruled out, as studies from other countries have noted the simultaneous contamination of chicken flocks by several genotypes [35,36].

There may be several reasons for the low prevalence of *C*. *jejuni* in Finnish slaughter batches, among these being the high level of biosecurity on Finnish chicken farms. Strict hygiene and biosecurity are suggested to be most successful measures against environmental contamination of chicken [37–40]. The close relationship between chicken producers and slaughterhouses has led to highly educated and aware farmers, which receive continuous training, guidance, and surveillance from slaughterhouse-employed veterinarians. Furthermore, the obligatory *Salmonella* control and *Campylobacter* monitoring program may have had a cumulative effect on strengthening the level of biosecurity (MMMa 1173/200, MMMa 10/EEO/2007)[14]. Because thinning is not used in Finland [13], the lack of this practice may also influence the *C*. *jejuni* prevalence in a favourable way since increased *C*. *jejuni* contamination risk has been found to be associated with partial depopulation practices [17,25].

Besides good biosecurity, climate may directly and/or indirectly help to keep the *C*. *jejuni* prevalence low in Finnish chickens. Finnish chicken farms are located in five counties in the southwest, western and central parts of the country where the average daily temperature is below 6°C for seven months of the year [41,42]. Temperatures below 6°C have been found to protect against *C*. *jejuni* contamination of chicken flocks [43], and the cold climate and snow coverage during an extended time period may therefore partly explain the extremely low *C*. *jejuni* prevalence in Finnish chicken flocks during winter [44]. Also, the low *C*. *jejuni* occurrence in Finnish chickens during winter minimizes the load of the contamination from chickens to the environment, thereby greatly reducing possible transmission to other hosts or vehicles. The cold climate has also necessitated building well-insulated chicken houses, which restricts the admission of rodents, pets and insects, further improving biosecurity.

From the *C*. *jejuni* isolated between 2004 and 2012, a total of 63 STs assigned into 12 CCs were found, indicating a high degree of diversity in the *C*. *jejuni* populations colonizing chicken flocks. Inclusion of PFGE genotyping in the analysis increased the detected diversity further. However, some MLST types, for instance ST-45 CC and ST-677 CC, dominated the population over time. This type of population structure in chicken-derived *C*. *jejuni*, i.e. a high degree of diversity coupled with a few predominant genotypes, seems to be typical among chicken flocks [23]. In our study we were also able to show that this population structure persisted over a long time period, nine years, and did not vary significantly from year to year. These predominating, persisting lineages detected from Finnish chickens have previously been isolated from a wide range of other hosts as well as environmental sources (http://pubmlst.org/campylobacter) [7,23,45,46]. However, a spatial effect on the relative contribution of different CCs and STs in the *C*. *jejuni* populations dependent on geographical area was also noted in our material [47]. For instance, every second and third Finnish chicken isolate was a member of ST-45 CC and ST-45, respectively. Contrary to this, Griekspoor et al. (2010) and Sheppard et al. (2009) [7,23] found approximately equal amounts of ST -45 CC and ST-21 CC, ST-257 CC, and ST-48 CC, among Swedish and Scottish chickens, respectively. Furthermore, the predominance of ST-45 CC and ST-45 has earlier been found to reflect the *C*. *jejuni* types of Finnish campylobacteriosis patients [22,47], suggesting that chicken may be a reservoir or source for the *C*. *jejuni* lineages infecting humans. Another hypothesis could be that a third shared, yet unidentified, source exists for both humans and chickens.

The exceptionally high frequency of ST-45 CC in our data could already be seen in 2003, as ST-45 dominated the MLST types found among *C*. *jejuni* isolates collected from Finnish chicken meat [46]. The exact reasons for the predominance of this lineage are not known; however, French et al. (2014) [48] proposed that geography and host cooperatively and independently act as determinants for the *C*. *jejuni* population structure. This effect may have contributed to the relatively high frequency of ST-45 CC in Finnish chickens, via factors yet unidentified in Finland. An additional possible explanation may lie in the seasonal influence on ST-45 CC described in the United Kingdom. McCarthy et al. (2012) [47] found an increasing number of the isolates belonging to this clonal complex during the summer months, but in our regression analysis no seasonal effect on the occurrence of ST-45 CC was seen. There was however increased odds of detecting ST-45 in September compared to spring in our study. September is part of the summer peak in Finland and since ST-45 is the major ST in the ST-45 CC our results seem to be in line with those of McCarthy et al. (2012) [47].

Another specific feature among the *C*. *jejuni* population was the high isolation frequency of ST-677 CC and ST-283 CC in Finnish chickens. Although both of these MLST types have been found in several hosts according to the pubmlst database (http://pubmlst.org/campylobacter), neither of them is among the frequently isolated CCs from chickens internationally [29,36,49]. Our data contained too few isolates of the ST-283 CC to draw any firm conclusions about the predictive effects of year, season, and site of slaughter, but the same seasonal effects acting on ST-45 CC have also been described for ST-283 CC [47]. This may be a plausible reason for the high frequency of ST-283 CC in Finnish chickens. ST-677, the main sequence type in the ST-677 CC, may have a special ecological advantage in adaptation in the Finnish chicken production chain, similar to ST-45 CC, and this clonal complex is also frequently found in Finnish campylobacteriosis patients [46]. According to our regression analyses, the odds for a *C*. *jejuni*-positive chicken flock to be of the ST-677 CC were equally distributed among years and sites of slaughter, and thus, the factors underlying the high prevalence of this lineage on Finnish chicken farms remains unknown.

Differences in the occurrence of ST-45 CC and ST-21 CC according to the site of slaughter, which could not be explained by the geographical location of the ten “high-frequency” farms, emerged in our study. More specifically, increased odds for detecting ST-45 CC was seen in Area A and C compared to Area B and higher odds for ST-21 CC was found in Areas B and C relative to Area A. These area codes reflect quite well the farm locations as Finnish chicken farms are located in close proximity to their respective slaughterhouse, but it is unlikely that different climatic conditions would have resulted in the observed selection pattern for the above-mentioned clonal complexes. Rather, it is plausible that differing environmental contamination patterns and farm management practices could be responsible for the selection of these clonal complexes, but more detailed environmental and husbandry data collected from these farms are needed to confirm this speculation.

One central finding was that most of the Finnish chicken farms, on average 72.2% annually, did not deliver any *C*. *jejuni*-positive batches. Furthermore, the majority of the positive farms (59.6%) delivered positive batches only in one of the study years. In addition, when multiple *C*. *jejuni*-positive batches originated from the same farm, nearly half of these farms had raised these chicken batches during the same rearing cycle (48.2% of the farms). This rare and sporadic contamination pattern of chicken flocks most probably results from a variety of effective hygiene barriers on Finnish farms, since *C*. *jejuni* only seldomly appeared to colonize the chickens. In addition, no indications of a persistent *C*. *jejuni* contamination source were found, as the few farms (n = 14) yielding the same ST over multiple years rarely had the same PFGE profile (14.3%). This was also the case for three of the ten “high-frequency” farms, from which ST-45 (Farms B1 and C5) and ST-677 (Farm C4) were recurrently isolated in consecutive years, but these isolates were all of different PFGE profiles. Our findings are in line with earlier studies suggesting that horizontal transmission originates from the surrounding environment and gains access to the chicken house via farm workers or other vectors (insects, rodents) [36,37,50]. However, the exact sources for *Campylobacter* in the surrounding environment have not been identified in most on-farm genotyping studies [24,51]. In addition, the lack of evidence for existing *C*. *jejuni* carry-over between different rearing cycles confirms the adequacy of the all-in all-out system practiced and that sufficient down-time and disinfection are used.

On the two exceptional farms in which the STs also had the same PFGE profile over subsequent years, ST-45/SmaI S7 was isolated in both 2004 and 2007 on Farm B10 and ST-45/SmaI S66 in 2006 and 2007 on Farm C14. The ST-45 is especially widespread and considered to be a generalist able to colonize several hosts [6]. The recurrence of such an abundant ST with its significantly associated PFGE genotypes might therefore reflect the introduction of a different strain of the same ST-45 to the chicken house the following year rather than the existence of a persistent contamination source to the chicken flocks on these farms.

Even though no persistent *C*. *jejuni* contamination source was evident between rearing cycles or different years, several of the positive farms delivered more than one positive batch during one rearing cycle (n = 55). These birds were raised in different chicken houses, and their *C*. *jejuni* isolates often had the same genotype (MLST, PFGE). This implies that transmission of the same *C*. *jejuni* clone has occurred between flocks in separate buildings, probably through workers or other vehicles. As noted in earlier studies, the risk of *Campylobacter* colonization increases with the number of houses on a farm [52] due to an accumulation of environmental *C*. *jejuni* contamination around the chicken houses [53]. *C*. *jejuni* may subsequently gain access to the neighboring chicken houses if there is a breach in biosecurity, so strict enforcement of hygiene barriers between houses is of especially important on multi-house farms.

In conclusion, the low occurrence of *C*. *jejuni*-positive chicken flocks in Finland reflects the presence of high-level biosecurity in combination with climatic conditions that supports virtually *Campylobacter*-free production cycles in the winter. However, each year a quarter of Finnish chicken farms deliver at least one positive slaughter batch. The *C*. *jejuni* population in Finnish chickens was overall genetically diverse, but certain predominating lineages exist (ST-45 CC, ST-677 CC, and ST-45) and may be associated with climate, geography, and an overrepresentation of these MLST types in environmental contamination sources. Furthermore, no evidence for a persistent *C*. *jejuni* source on Finnish chicken farms emerged, and *C*. *jejuni* colonization of chicken flocks are probably introduced during the rearing period from the surrounding area.

## Supporting Information

S1 Table Overview of the number of clonal complexes (CC) and sequence types (ST) isolated from Finnish broiler batches according to year. Frequencies from the non-adjusted and adjusted database are given.
(DOCX)

S2 Table Overview of the number of clonal complexes (CC) and sequence types (ST) isolated from Finnish broiler batches according to month of collection. Frequencies from the non-adjusted and adjusted database are given.
(DOCX)

S3 Table Overview of the Pulsed-field gel electrophoresis SmaI and KpnI types found in Finnish broilers in 2004, 2006, 2007, 2008 and 2012. All 363 PFGE typed isolates are included in the table.
(DOCX)
