# Supplementary material for: Predominant Campylobacter jejuni Sequence Types Persist in Finnish Chicken Production
Source: PLoS One. 2015 Feb 20;10(2):e0116585. doi: 10.1371/journal.pone.0116585 (PMC4336332; doi:10.1371/journal.pone.0116585)
Supplement: S1 Table — Frequencies from the non-adjusted and adjusted database are given. (DOCX) [file pone.0116585.s001.docx]

**S1 Table.** Overview over number of clonal complexes (CC) and sequence types (ST) isolated from Finnish broiler batches according to year. Frequencies from the non-adjusted and adjusted database are given.

|  | **Year** | | | | |  |
| --- | --- | --- | --- | --- | --- | --- |
| **CC** | **2004** | **2006** | **2007** | **2008** | **2012** | **Total** |
| 1034 | 0/0^A^ | 0/0^A^ | 3/3^A^ | 0/0^A^ | 2/1^A^ | 5/4^A^ |
| 1287 | 0/0^A^ | 4/2^A^ | 0/0^A^ | 0/0^A^ | 1/1^A^ | 5/3^A^ |
| 1332 | 0/0^A^ | 0/0^A^ | 5/3^A^ | 0/0^A^ | 1/1^A^ | 6/4^A^ |
| 177 | 1/1^A^ | 0/0^A^ | 0/0^A^ | 0/0^A^ | 0/0^A^ | 1/1^A^ |
| 21 | 6/5^A^ | 6/4^A^ | 6/4^A^ | 22/12^A^ | 0/0^A^ | 40/25^A^ |
| 283 | 0/0^A^ | 10/7^A^ | 3/2^A^ | 1/1^A^ | 11/5^A^ | 25/15^A^ |
| 353 | 0/0^A^ | 0/0^A^ | 3/3^A^ | 0/0^A^ | 0/0^A^ | 3/3^A^ |
| 42 | 0/0^A^ | 0/0^A^ | 0/0^A^ | 0/0^A^ | 1/1^A^ | 1/1^A^ |
| 45 | 43/29^A^ | 29/24^A^ | 41/25^A^ | 35/30^A^ | 51/38^A^ | 199/146^A^ |
| 677 | 5/5^A^ | 5/4^A^ | 14/9^A^ | 8/5^A^ | 7/6^A^ | 39/29^A^ |
| 692 | 0/0^A^ | 0/0^A^ | 2/1^A^ | 3/2^A^ | 0/0^A^ | 5/3 |
| 952 | 0/0^A^ | 0/0^A^ | 0/0^A^ | 1/1^A^ | 1/1^A^ | 2/2^A^ |
| UA | 14/11^A^ | 6/6^A^ | 11/9^A^ | 8/7^A^ | 8/4^A^ | 47/37^A^ |
| **Total** | **69/51^A^** | **60/47^A^** | **88/59^A^** | **78/58^A^** | **83/58^A^** | **378/273^A^** |
| **ST** | 2004 | 2006 | 2007 | 2008 | 2012 | **Total** |
| 1003 | 5/1^A^ | 0/0^A^ | 0/0^A^ | 1/1^A^ | 0/0^A^ | 6/2^A^ |
| 1080 | 0/0^A^ | 0/0^A^ | 0/0^A^ | 1/1^A^ | 0/0^A^ | 1/1^A^ |
| 11 | 1/1^A^ | 1/1^A^ | 0/0^A^ | 3/3^A^ | 10/7^A^ | 15/12^A^ |
| 1276 | 0/0^A^ | 0/0^A^ | 0/0^A^ | 0/0^A^ | 1/1^A^ | 1/1^A^ |
| 1278 | 0/0^A^ | 0/0^A^ | 2/1^A^ | 0/0^A^ | 0/0^A^ | 2/1^A^ |
| 1326 | 3/1^A^ | 0/0^A^ | 5/3^A^ | 4/2^A^ | 0/0^A^ | 12/6^A^ |
| 1332 | 0/0^A^ | 0/0^A^ | 3/1^A^ | 0/0^A^ | 0/0^A^ | 3/1^A^ |
| 1367 | 2/1^A^ | 1/1^A^ | 2/2^A^ | 2/2^A^ | 0/0^A^ | 7/6 ^A^ |
| 137 | 0/0^A^ | 1/1^A^ | 0/0^A^ | 3/2^A^ | 0/0^A^ | 4/3^A^ |
| 1539 | 1/1^A^ | 0/0^A^ | 0/0^A^ | 0/0^A^ | 0/0^A^ | 1/1^A^ |
| 1721 | 0/0^A^ | 0/0^A^ | 0/0^A^ | 0/0^A^ | 2/1^A^ | 2/1^A^ |
| 1970 | 1/1^A^ | 0/0^A^ | 0/0^A^ | 0/0^A^ | 0/0^A^ | 1/1^A^ |
| 2046 | 0/0^A^ | 1/1^A^ | 0/0^A^ | 0/0^A^ | 0/0^A^ | 1/1^A^ |
| 2219 | 0/0^A^ | 0/0^A^ | 3/3^A^ | 2/2^A^ | 0/0^A^ | 5/5^A^ |
| 230 | 11/7^A^ | 0/0^A^ | 1/1^A^ | 4/4^A^ | 3/3^A^ | 19/15^A^ |
| 267 | 0/0^A^ | 10/7^A^ | 3/2^A^ | 1/1^A^ | 11/5^A^ | 25/15^A^ |
| 2856 | 1/1^A^ | 0/0^A^ | 0/0^A^ | 0/0^A^ | 0/0^A^ | 1/1^A^ |
| 3128 | 1/1^A^ | 0/0^A^ | 0/0^A^ | 0/0^A^ | 0/0^A^ | 1/1^A^ |
| 3272 | 0/0^A^ | 0/0^A^ | 3/1^A^ | 0/0^A^ | 4/1^A^ | 7/2^A^ |
| 334 | 0/0^A^ | 0/0^A^ | 1/1^A^ | 0/0^A^ | 0/0^A^ | 1/1^A^ |
| 3449 | 0/0^A^ | 1/1^A^ | 0/0^A^ | 0/0^A^ | 0/0^A^ | 1/1^A^ |
| 3453 | 0/0^A^ | 1/1^A^ | 0/0^A^ | 0/0^A^ | 0/0^A^ | 1/1^A^ |
| 3477 | 1/1^A^ | 0/0^A^ | 0/0^A^ | 0/0^A^ | 0/0^A^ | 1/1^A^ |
| 3502 | 0/0^A^ | 0/0^A^ | 1/1^A^ | 0/0^A^ | 0/0^A^ | 1/1^A^ |
| 356 | 0/0^A^ | 0/0^A^ | 3/3^A^ | 0/0^A^ | 0/0^A^ | 3/3^A^ |
| 3755 | 0/0^A^ | 0/0^A^ | 1/1^A^ | 0/0^A^ | 0/0^A^ | 1/1^A^ |
| 3791 | 0/0^A^ | 0/0^A^ | 1/1^A^ | 0/0^A^ | 0/0^A^ | 1/1^A^ |
| 3805 | 0/0^A^ | 0/0^A^ | 8/4^A^ | 0/0^A^ | 0/0^A^ | 8/4^A^ |
| 3865 | 1/1^A^ | 0/0^A^ | 0/0^A^ | 0/0^A^ | 0/0^A^ | 1/1^A^ |
| 3999 | 0/0^A^ | 1/1^A^ | 0/0^A^ | 0/0^A^ | 0/0^A^ | 1/1^A^ |
| 4000 | 0/0^A^ | 1/1^A^ | 0/0^A^ | 0/0^A^ | 0/0^A^ | 1/1^A^ |
| 4001 | 0/0^A^ | 0/0^A^ | 3/3^A^ | 0/0^A^ | 2/1^A^ | 5/4^A^ |
| 4002 | 0/0^A^ | 0/0^A^ | 2/2^A^ | 0/0^A^ | 0/0^A^ | 2/2^A^ |
| 4003 | 0/0^A^ | 0/0^A^ | 1/1^A^ | 0/0^A^ | 0/0^A^ | 1/1^A^ |
| 4004 | 0/0^A^ | 0/0^A^ | 1/1^A^ | 0/0^A^ | 0/0^A^ | 1/1^A^ |
| 42 | 0/0^A^ | 0/0^A^ | 0/0^A^ | 0/0^A^ | 1/1^A^ | 1/1^A^ |
| 4307 | 0/0^A^ | 0/0^A^ | 0/0^A^ | 2/1^A^ | 0/0^A^ | 2/1^A^ |
| 448 | 0/0^A^ | 0/0^A^ | 1/1^A^ | 0/0^A^ | 0/0^A^ | 1/1^A^ |
| 45 | 22/18^A^ | 23/18^A^ | 20/11^A^ | 18/16^A^ | 36/27^A^ | 119/90^A^ |
| 451 | 2/2^A^ | 2/2^A^ | 0/0^A^ | 19/9^A^ | 0/0^A^ | 23/13^A^ |
| 4596 | 2/2^A^ | 0/0^A^ | 0/0^A^ | 0/0^A^ | 0/0^A^ | 2/2^A^ |
| 50 | 1/1^A^ | 4/2^A^ | 3/2^A^ | 3/3^A^ | 0/0^A^ | 11/8^A^ |
| 5201 | 1/1^A^ | 0/0^A^ | 0/0^A^ | 0/0^A^ | 0/0^A^ | 1/1^A^ |
| 53 | 2/1^A^ | 0/0^A^ | 3/2^A^ | 0/0^A^ | 0/0^A^ | 5/3^A^ |
| 538 | 0/0^A^ | 0/0^A^ | 2/1^A^ | 0/0^A^ | 0/0^A^ | 2/1^A^ |
| 5528 | 1/1^A^ | 0/0^A^ | 0/0^A^ | 0/0^A^ | 0/0^A^ | 1/1^A^ |
| 583 | 0/0^A^ | 3/3 ^A^ | 0/0^A^ | 0/0^A^ | 2/1^A^ | 5/4 ^A^ |
| 586 | 0/0^A^ | 1/1^A^ | 0/0^A^ | 0/0^A^ | 0/0^A^ | 1/1^A^ |
| 6228 | 0/0^A^ | 0/0^A^ | 0/0^A^ | 1/1^A^ | 0/0^A^ | 1/1^A^ |
| 6236 | 1/1^A^ | 0/0^A^ | 0/0^A^ | 0/0^A^ | 0/0^A^ | 1/1^A^ |
| 6237 | 1/1^A^ | 0/0^A^ | 0/0^A^ | 0/0^A^ | 0/0^A^ | 1/1^A^ |
| 6460 | 0/0^A^ | 0/0^A^ | 0/0^A^ | 0/0^A^ | 1/1^A^ | 1/1^A^ |
| 6471 | 0/0^A^ | 0/0^A^ | 0/0^A^ | 0/0^A^ | 1/1^A^ | 1/1^A^ |
| 6555 | 0/0^A^ | 0/0^A^ | 0/0^A^ | 0/0^A^ | 1/1^A^ | 1/1^A^ |
| 6556 | 0/0^A^ | 0/0^A^ | 0/0^A^ | 0/0^A^ | 1/1^A^ | 1/1^A^ |
| 677 | 5/5^A^ | 5/4^A^ | 13/8^A^ | 8/5^A^ | 4/4^A^ | 35/26^A^ |
| 692 | 0/0^A^ | 0/0^A^ | 0/0^A^ | 3/2^A^ | 0/0^A^ | 3/2^A^ |
| 7008 | 3/1^A^ | 0/0^A^ | 0/0^A^ | 0/0^A^ | 0/0^A^ | 3/1^A^ |
| 7011 | 0/0^A^ | 0/0^A^ | 0/0^A^ | 1/1^A^ | 0/0^A^ | 1/1^A^ |
| 7020 | 0/0^A^ | 0/0^A^ | 0/0^A^ | 2/2^A^ | 0/0^A^ | 2/2^A^ |
| 794 | 0/0^A^ | 0/0^A^ | 1/1^A^ | 0/0^A^ | 3/2^A^ | 4/3^A^ |
| 945 | 0/0^A^ | 4/2^A^ | 0/0^A^ | 0/0^A^ | 0/0^A^ | 4/2^A^ |
| 993 | 0/0^A^ | 0/0^A^ | 1/1^A^ | 0/0^A^ | 0/0^A^ | 1/1^A^ |
| **Total** | **69/51^A^** | **60/47^A^** | **88/59^A^** | **78/58^A^** | **83/58^A^** | **378/273^A^** |

^A^ Frequency is calculated from the adjusted database, meaning that isolates originating from the same farm on the same date with identical MLST and PFGE types accounts for one isolate.
