# Supplementary material for: Predominant Campylobacter jejuni Sequence Types Persist in Finnish Chicken Production
Source: PLoS One. 2015 Feb 20;10(2):e0116585. doi: 10.1371/journal.pone.0116585 (PMC4336332; doi:10.1371/journal.pone.0116585)
Supplement: S2 Table — Frequencies from the non-adjusted and adjusted database are given. (DOCX) [file pone.0116585.s002.docx]

**Table S2.** Overview over number of clonal complexes (CC) and sequence types (ST) isolated from Finnish broiler batches according to month of collection. Frequencies from the non-adjusted and adjusted database are given.

|  |  | | **Month** | | | | | | | | |  |
| --- | --- | --- | --- | --- | --- | --- | --- | --- | --- | --- | --- | --- |
| **CC** | March | April | | May | June | July | August | Sept | Oct | Nov | Dec | Total |
| 1034 | 0/0^A^ | 0/0^A^ | | 0/0^A^ | 0/0^A^ | 2/2^A^ | 3/2 ^A^ | 0/0 ^A^ | 0/0^A^ | 0/0^A^ | 0/0^A^ | 5/4^A^ |
| 1287 | 0/0^A^ | 0/0^A^ | | 0/0^A^ | 0/0^A^ | 3/2^A^ | 2/1^A^ | 0/0^A^ | 0/0^A^ | 0/0^A^ | 0/0^A^ | 5/3^A^ |
| 1332 | 0/0^A^ | 0/0^A^ | | 1/1 ^A^ | 0/0^A^ | 5/3^A^ | 0/0^A^ | 0/0^A^ | 0/0^A^ | 0/0^A^ | 0/0^A^ | 6/4^A^ |
| 177 | 0/0^A^ | 0/0^A^ | | 0/0^A^ | 0/0^A^ | 0/0^A^ | 0/0^A^ | 1/1 ^A^ | 0/0^A^ | 0/0^A^ | 0/0^A^ | 1/1^A^ |
| 21 | 0/0^A^ | 0/0^A^ | | 0/0^A^ | 5/5^A^ | 10/6^A^ | 18/10^A^ | 5/3^A^ | 2/1^A^ | 0/0^A^ | 0/0^A^ | 40/25^A^ |
| 283 | 0/0^A^ | 0/0^A^ | | 0/0^A^ | 1/1^A^ | 14/8^A^ | 7/4^A^ | 3/2^A^ | 0/0^A^ | 0/0^A^ | 0/0^A^ | 25/15^A^ |
| 353 | 0/0^A^ | 0/0^A^ | | 0/0^A^ | 0/0^A^ | 0/0^A^ | 2/2^A^ | 1/1 ^A^ | 0/0^A^ | 0/0^A^ | 0/0^A^ | 3/3^A^ |
| 42 | 0/0^A^ | 0/0^A^ | | 0/0^A^ | 1/1^A^ | 0/0^A^ | 0/0^A^ | 0/0^A^ | 0/0^A^ | 0/0^A^ | 0/0^A^ | 1/1 ^A^ |
| 45 | 2/1^A^ | 1/1^A^ | | 0/0^A^ | 5/5^A^ | 83/61^A^ | 53/39^A^ | 33/25^A^ | 20/12^A^ | 1/1^A^ | 1/1^A^ | 199/146^A^ |
| 677 | 0/0^A^ | 0/0^A^ | | 0/0^A^ | 0/0^A^ | 9/6^A^ | 25/20^A^ | 5/3^A^ | 0/0^A^ | 0/0^A^ | 0/0^A^ | 39/29^A^ |
| 692 | 0/0^A^ | 0/0^A^ | | 0/0^A^ | 0/0^A^ | 0/0^A^ | 1/1^A^ | 4/2^A^ | 0/0^A^ | 0/0^A^ | 0/0^A^ | 5/3^A^ |
| 952 | 0/0^A^ | 0/0^A^ | | 0/0^A^ | 0/0^A^ | 1/1 ^A^ | 1/1^A^ | 0/0^A^ | 0/0^A^ | 0/0^A^ | 0/0^A^ | 2/2^A^ |
| UA | 0/0^A^ | 0/0^A^ | | 0/0^A^ | 2/2^A^ | 16/12^A^ | 12/9^A^ | 5/5^A^ | 9/7^A^ | 3/2^A^ | 0/0^A^ | 47/37^A^ |
| **Total** | **2/1^A^** | **1/1^A^** | | **1/1^A^** | **14/14^A^** | **143/101^A^** | **124/89^A^** | **57/42^A^** | **31/20^A^** | **4/3^A^** | **1/1^A^** | **378/273^A^** |
| **ST** | March | April | | May | June | July | August | Sept | Oct | Nov | Dec | Total |
| 1003 | 0/0^A^ | 0/0^A^ | | 0/0^A^ | 0/0^A^ | 1/1^A^ | 5/1^A^ | 0/0^A^ | 0/0^A^ | 0/0^A^ | 0/0^A^ | 6/2^A^ |
| 1080 | 0/0^A^ | 0/0^A^ | | 0/0^A^ | 0/0^A^ | 1/1^A^ | 0/0^A^ | 0/0^A^ | 0/0^A^ | 0/0^A^ | 0/0^A^ | 1/1^A^ |
| 11 | 0/0^A^ | 0/0^A^ | | 0/0^A^ | 1/1^A^ | 5/5^A^ | 9/6^A^ | 0/0^A^ | 0/0^A^ | 0/0^A^ | 0/0^A^ | 15/12^A^ |
| 1276 | 0/0^A^ | 0/0^A^ | | 1/1^A^ | 0/0^A^ | 0/0^A^ | 0/0^A^ | 0/0^A^ | 0/0^A^ | 0/0^A^ | 0/0^A^ | 1/1^A^ |
| 1278 | 0/0^A^ | 0/0^A^ | | 0/0^A^ | 0/0^A^ | 0/0^A^ | 0/0^A^ | 2/1^A^ | 0/0^A^ | 0/0^A^ | 0/0^A^ | 2/1^A^ |
| 1326 | 0/0^A^ | 0/0^A^ | | 0/0^A^ | 0/0^A^ | 2/2^A^ | 6/2^A^ | 4/2^A^ | 0/0^A^ | 0/0^A^ | 0/0^A^ | 12/6^A^ |
| 1332 | 0/0^A^ | 0/0^A^ | | 0/0^A^ | 0/0^A^ | 3/1^A^ | 0/0^A^ | 0/0^A^ | 0/0^A^ | 0/0^A^ | 0/0^A^ | 3/1^A^ |
| 1367 | 0/0^A^ | 0/0^A^ | | 0/0^A^ | 1/1^A^ | 3/3^A^ | 3/2^A^ | 0/0^A^ | 0/0^A^ | 0/0^A^ | 0/0^A^ | 7/6^A^ |
| 137 | 0/0^A^ | 0/0^A^ | | 0/0^A^ | 0/0^A^ | 1/1^A^ | 3/2^A^ | 0/0^A^ | 0/0^A^ | 0/0^A^ | 0/0^A^ | 4/3^A^ |
| 1539 | 0/0^A^ | 0/0^A^ | | 0/0^A^ | 0/0^A^ | 0/0^A^ | 1/1^A^ | 0/0^A^ | 0/0^A^ | 0/0^A^ | 0/0^A^ | 1/1^A^ |
| 1721 | 0/0^A^ | 0/0^A^ | | 0/0^A^ | 0/0^A^ | 2/1^A^ | 0/0^A^ | 0/0^A^ | 0/0^A^ | 0/0^A^ | 0/0^A^ | 2/1^A^ |
| 1970 | 0/0^A^ | 0/0^A^ | | 0/0^A^ | 0/0^A^ | 1/1^A^ | 0/0^A^ | 0/0^A^ | 0/0^A^ | 0/0^A^ | 0/0^A^ | 1/1^A^ |
| 2046 | 0/0^A^ | 0/0^A^ | | 0/0^A^ | 0/0^A^ | 1/1^A^ | 0/0^A^ | 0/0^A^ | 0/0^A^ | 0/0^A^ | 0/0^A^ | 1/1^A^ |
| 2219 | 0/0^A^ | 0/0^A^ | | 0/0^A^ | 0/0^A^ | 0/0^A^ | 5/5^A^ | 0/0^A^ | 0/0^A^ | 0/0^A^ | 0/0^A^ | 5/5^A^ |
| 230 | 0/0^A^ | 0/0^A^ | | 0/0^A^ | 0/0^A^ | 10/7^A^ | 7/7^A^ | 2/1^A^ | 0/0^A^ | 0/0^A^ | 0/0^A^ | 19/15^A^ |
| 267 | 0/0^A^ | 0/0^A^ | | 0/0^A^ | 1/1^A^ | 14/8^A^ | 7/4^A^ | 3/2^A^ | 0/0^A^ | 0/0^A^ | 0/0^A^ | 25/15^A^ |
| 2856 | 0/0^A^ | 0/0^A^ | | 0/0^A^ | 0/0^A^ | 0/0^A^ | 0/0^A^ | 0/0^A^ | 1/1^A^ | 0/0^A^ | 0/0^A^ | 1/1^A^ |
| 3128 | 0/0^A^ | 0/0^A^ | | 0/0^A^ | 1/1^A^ | 0/0^A^ | 0/0^A^ | 0/0^A^ | 0/0^A^ | 0/0^A^ | 0/0^A^ | 1/1^A^ |
| 3272 | 0/0^A^ | 0/0^A^ | | 0/0^A^ | 0/0^A^ | 4/1^A^ | 3/1^A^ | 0/0^A^ | 0/0^A^ | 0/0^A^ | 0/0^A^ | 7/2 |
| 334 | 0/0^A^ | 0/0^A^ | | 0/0^A^ | 0/0^A^ | 0/0^A^ | 1/1^A^ | 0/0^A^ | 0/0^A^ | 0/0^A^ | 0/0^A^ | 1/1^A^ |
| 3449 | 0/0^A^ | 0/0^A^ | | 0/0^A^ | 0/0^A^ | 1/1^A^ | 0/0^A^ | 0/0^A^ | 0/0^A^ | 0/0^A^ | 0/0^A^ | 1/1^A^ |
| 3453 | 0/0^A^ | 0/0^A^ | | 0/0^A^ | 0/0^A^ | 0/0^A^ | 1/1^A^ | 0/0^A^ | 0/0^A^ | 0/0^A^ | 0/0^A^ | 1/1^A^ |
| 3477 | 0/0^A^ | 0/0^A^ | | 0/0^A^ | 0/0^A^ | 0/0^A^ | 1/1^A^ | 0/0^A^ | 0/0^A^ | 0/0^A^ | 0/0^A^ | 1/1^A^ |
| 3502 | 0/0^A^ | 0/0^A^ | | 0/0^A^ | 0/0^A^ | 1/1^A^ | 0/0^A^ | 0/0^A^ | 0/0^A^ | 0/0^A^ | 0/0^A^ | 1/1^A^ |
| 356 | 0/0^A^ | 0/0^A^ | | 0/0^A^ | 0/0^A^ | 0/0^A^ | 2/2^A^ | 1/1^A^ | 0/0^A^ | 0/0^A^ | 0/0^A^ | 3/3^A^ |
| 3755 | 0/0^A^ | 0/0^A^ | | 0/0^A^ | 0/0^A^ | 1/1^A^ | 0/0^A^ | 0/0^A^ | 0/0^A^ | 0/0^A^ | 0/0^A^ | 1/1^A^ |
| 3791 | 0/0^A^ | 0/0^A^ | | 0/0^A^ | 0/0^A^ | 0/0^A^ | 1/1^A^ | 0/0^A^ | 0/0^A^ | 0/0^A^ | 0/0^A^ | 1/1^A^ |
| 3805 | 0/0^A^ | 0/0^A^ | | 0/0^A^ | 1/1^A^ | 7/3^A^ | 0/0^A^ | 0/0^A^ | 0/0^A^ | 0/0^A^ | 0/0^A^ | 8/4^A^ |
| 3865 | 0/0^A^ | 0/0^A^ | | 0/0^A^ | 0/0^A^ | 0/0^A^ | 0/0^A^ | 1/1^A^ | 0/0^A^ | 0/0^A^ | 0/0^A^ | 1/1^A^ |
| 3999 | 0/0^A^ | 0/0^A^ | | 0/0^A^ | 1/1^A^ | 0/0^A^ | 0/0^A^ | 0/0^A^ | 0/0^A^ | 0/0^A^ | 0/0^A^ | 1/1^A^ |
| 4000 | 0/0^A^ | 0/0^A^ | | 0/0^A^ | 0/0^A^ | 0/0^A^ | 0/0^A^ | 0/0^A^ | 1/1^A^ | 0/0^A^ | 0/0^A^ | 1/1^A^ |
| 4001 | 0/0^A^ | 0/0^A^ | | 0/0^A^ | 0/0^A^ | 2/2^A^ | 3/2^A^ | 0/0^A^ | 0/0^A^ | 0/0^A^ | 0/0^A^ | 5/4^A^ |
| 4002 | 0/0^A^ | 0/0^A^ | | 0/0^A^ | 0/0^A^ | 2/2^A^ | 0/0^A^ | 0/0^A^ | 0/0^A^ | 0/0^A^ | 0/0^A^ | 2/2^A^ |
| 4003 | 0/0^A^ | 0/0^A^ | | 0/0^A^ | 0/0^A^ | 0/0^A^ | 0/0^A^ | 0/0^A^ | 1/1^A^ | 0/0^A^ | 0/0^A^ | 1/1^A^ |
| 4004 | 0/0^A^ | 0/0^A^ | | 0/0^A^ | 0/0^A^ | 1/1^A^ | 0/0^A^ | 0/0^A^ | 0/0^A^ | 0/0^A^ | 0/0^A^ | 1/1^A^ |
| 42 | 0/0^A^ | 0/0^A^ | | 0/0^A^ | 1/1^A^ | 0/0^A^ | 0/0^A^ | 0/0^A^ | 0/0^A^ | 0/0^A^ | 0/0^A^ | 1/1^A^ |
| 4307 | 0/0^A^ | 0/0^A^ | | 0/0^A^ | 0/0^A^ | 0/0^A^ | 0/0^A^ | 0/0^A^ | 0/0^A^ | 2/1^A^ | 0/0^A^ | 2/1^A^ |
| 448 | 0/0^A^ | 0/0^A^ | | 0/0^A^ | 0/0^A^ | 0/0^A^ | 0/0^A^ | 0/0^A^ | 1/1^A^ | 0/0^A^ | 0/0^A^ | 1/1^A^ |
| 45 | 2/1^A^ | 1/1^A^ | | 0/0^A^ | 2/2^A^ | 54/39^A^ | 13/12^A^ | 25/21^A^ | 20/12^A^ | 1/1^A^ | 1/1^A^ | 119/90^A^ |
| 451 | 0/0^A^ | 0/0^A^ | | 0/0^A^ | 1/1^A^ | 9/5^A^ | 11/5^A^ | 2/2^A^ | 0/0^A^ | 0/0^A^ | 0/0^A^ | 23/13^A^ |
| 4596 | 0/0^A^ | 0/0^A^ | | 0/0^A^ | 0/0^A^ | 0/0^A^ | 0/0^A^ | 0/0^A^ | 1/1^A^ | 1/1^A^ | 0/0^A^ | 2/2^A^ |
| 50 | 0/0^A^ | 0/0^A^ | | 0/0^A^ | 3/3^A^ | 1/1^A^ | 4/3^A^ | 3/1^A^ | 0/0^A^ | 0/0^A^ | 0/0^A^ | 11/8^A^ |
| 5201 | 0/0^A^ | 0/0^A^ | | 0/0^A^ | 0/0^A^ | 0/0^A^ | 1/1^A^ | 0/0^A^ | 0/0^A^ | 0/0^A^ | 0/0^A^ | 1/1^A^ |
| 53 | 0/0^A^ | 0/0^A^ | | 0/0^A^ | 0/0^A^ | 0/0^A^ | 3/2^A^ | 0/0^A^ | 2/0^A^ | 0/0^A^ | 0/0^A^ | 5/3^A^ |
| 538 | 0/0^A^ | 0/0^A^ | | 0/0^A^ | 0/0^A^ | 2/1^A^ | 0/0^A^ | 0/0^A^ | 0/0^A^ | 0/0^A^ | 0/0^A^ | 2/1^A^ |
| 5528 | 0/0^A^ | 0/0^A^ | | 0/0^A^ | 0/0^A^ | 0/0^A^ | 0/0^A^ | 1/1^A^ | 0/0^A^ | 0/0^A^ | 0/0^A^ | 1/1^A^ |
| 583 | 0/0^A^ | 0/0^A^ | | 0/0^A^ | 1/1^A^ | 1/1^A^ | 1/1^A^ | 2/1^A^ | 1/1^A^ | 0/0^A^ | 0/0^A^ | 5/4^A^ |
| 586 | 0/0^A^ | 0/0^A^ | | 0/0^A^ | 0/0^A^ | 0/0^A^ | 0/0^A^ | 1/1^A^ | 0/0^A^ | 0/0^A^ | 0/0^A^ | 1/1^A^ |
| 6228 | 0/0^A^ | 0/0^A^ | | 0/0^A^ | 0/0^A^ | 1/1^A^ | 0/0^A^ | 0/0^A^ | 0/0^A^ | 0/0^A^ | 0/0^A^ | 1/1^A^ |
| 6236 | 0/0^A^ | 0/0^A^ | | 0/0^A^ | 0/0^A^ | 0/0^A^ | 1/1^A^ | 0/0^A^ | 0/0^A^ | 0/0^A^ | 0/0^A^ | 1/1^A^ |
| 6237 | 0/0^A^ | 0/0^A^ | | 0/0^A^ | 0/0^A^ | 0/0^A^ | 0/0^A^ | 1/1^A^ | 0/0^A^ | 0/0^A^ | 0/0^A^ | 1/1^A^ |
| 6460 | 0/0^A^ | 0/0^A^ | | 0/0^A^ | 0/0^A^ | 0/0^A^ | 0/0^A^ | 0/0^A^ | 1/1^A^ | 0/0^A^ | 0/0^A^ | 1/1^A^ |
| 6471 | 0/0^A^ | 0/0^A^ | | 0/0^A^ | 0/0^A^ | 0/0^A^ | 1/1^A^ | 0/0^A^ | 0/0^A^ | 0/0^A^ | 0/0^A^ | 1/1^A^ |
| 6555 | 0/0^A^ | 0/0^A^ | | 0/0^A^ | 0/0^A^ | 1/1^A^ | 0/0^A^ | 0/0^A^ | 0/0^A^ | 0/0^A^ | 0/0^A^ | 1/1^A^ |
| 6556 | 0/0^A^ | 0/0^A^ | | 0/0^A^ | 0/0^A^ | 0/0^A^ | 1/1^A^ | 0/0^A^ | 0/0^A^ | 0/0^A^ | 0/0^A^ | 1/1^A^ |
| 677 | 0/0^A^ | 0/0^A^ | | 0/0^A^ | 0/0^A^ | 7/5^A^ | 23/18^A^ | 5/3^A^ | 0/0^A^ | 0/0^A^ | 0/0^A^ | 35/26^A^ |
| 692 | 0/0^A^ | 0/0^A^ | | 0/0^A^ | 0/0^A^ | 0/0^A^ | 1/1^A^ | 2/1^A^ | 0/0^A^ | 0/0^A^ | 0/0^A^ | 3/^A^ |
| 7008 | 0/0^A^ | 0/0^A^ | | 0/0^A^ | 0/0^A^ | 0/0^A^ | 0/0^A^ | 0/0^A^ | 3/1^A^ | 0/0^A^ | 0/0^A^ | 3/1^A^ |
| 7011 | 0/0^A^ | 0/0^A^ | | 0/0^A^ | 0/0^A^ | 0/0^A^ | 1/1^A^ | 0/0^A^ | 0/0^A^ | 0/0^A^ | 0/0^A^ | 1/1^A^ |
| 7020 | 0/0^A^ | 0/0^A^ | | 0/0^A^ | 0/0^A^ | 0/0^A^ | 0/0^A^ | 2/2^A^ | 0/0^A^ | 0/0^A^ | 0/0^A^ | 2/2^A^ |
| 794 | 0/0^A^ | 0/0^A^ | | 0/0^A^ | 0/0^A^ | 2/1^A^ | 2/2^A^ | 0/0^A^ | 0/0^A^ | 0/0^A^ | 0/0^A^ | 4/3^A^ |
| 945 | 0/0^A^ | 0/0^A^ | | 0/0^A^ | 0/0^A^ | 2/1^A^ | 2/1^A^ | 0/0^A^ | 0/0^A^ | 0/0^A^ | 0/0^A^ | 4/2^A^ |
| 993 | 0/0^A^ | 0/0^A^ | | 0/0^A^ | 0/0^A^ | 1/1^A^ | 0/0^A^ | 0/0^A^ | 0/0^A^ | 0/0^A^ | 0/0^A^ | 1/1^A^ |
| **Total** | **2/1^A^** | **1/1^A^** | | **1/1^A^** | **14/14^A^** | **143/101^A^** | **124/89^A^** | **57/42^A^** | **31/20^A^** | **4/3^A^** | **1/1^A^** | **378/273^A^** |

^A^ Frequency is calculated from the adjusted database, meaning that isolates originating from the same farm on the same date with identical MLST and PFGE types accounts for one isolate.
