# Supplementary material for: Predominant Campylobacter jejuni Sequence Types Persist in Finnish Chicken Production
Source: PLoS One. 2015 Feb 20;10(2):e0116585. doi: 10.1371/journal.pone.0116585 (PMC4336332; doi:10.1371/journal.pone.0116585)
Supplement: S3 Table — All 363 PFGE typed isolates are included in the table. (DOCX) [file pone.0116585.s003.docx]

**Table S3.** Overview of Pulsed-field gel electrophoresis SmaI and KpnI types found in Finnish broilers in 2004, 2006, 2007, 2008 and 2012. All 363 PFGE typed isolates are included in the table.

| **FGE** | **Year** | | | | | **Total** |
| --- | --- | --- | --- | --- | --- | --- |
|  | 2004 | 2006 | 2007 | 2008 | 2012 |  |
| **K27** | 1 | 0 | 0 | 0 | 0 | 1 |
| **K36** | 10 | 9 | 4 | 2 | 10 | 35 |
| **K51** | 0 | 1 | 0 | 0 | 0 | 1 |
| **K53** | 1 | 0 | 0 | 0 | 0 | 1 |
| **K57** | 0 | 0 | 0 | 1 | 0 | 1 |
| **K59** | 1 | 0 | 0 | 0 | 0 | 1 |
| **K60** | 2 | 0 | 0 | 0 | 0 | 2 |
| **K61** | 0 | 0 | 0 | 1 | 0 | 1 |
| **K73** | 0 | 1 | 0 | 0 | 0 | 1 |
| **K74** | 0 | 0 | 0 | 0 | 2 | 2 |
| **K78** | 2 | 0 | 0 | 0 | 0 | 2 |
| **K85** | 0 | 0 | 2 | 0 | 0 | 2 |
| **S1** | 2 | 0 | 2 | 0 | 0 | 4 |
| **S101** | 0 | 1 | 0 | 0 | 0 | 1 |
| **S102** | 0 | 2 | 1 | 0 | 0 | 3 |
| **S103** | 0 | 0 | 1 | 0 | 0 | 1 |
| **S104** | 0 | 0 | 2 | 0 | 0 | 2 |
| **S105** | 0 | 3 | 0 | 0 | 0 | 3 |
| **S106** | 2 | 0 | 2 | 2 | 0 | 6 |
| **S108** | 0 | 0 | 2 | 0 | 0 | 2 |
| **S11** | 1 | 0 | 1 | 0 | 0 | 2 |
| **S110** | 0 | 0 | 9 | 0 | 0 | 9 |
| **S113** | 0 | 0 | 1 | 0 | 0 | 1 |
| **S116** | 0 | 0 | 3 | 0 | 0 | 3 |
| **S117** | 0 | 0 | 1 | 0 | 0 | 1 |
| **S118** | 0 | 0 | 1 | 0 | 0 | 1 |
| **S119** | 1 | 0 | 1 | 0 | 2 | 4 |
| **S12** | 0 | 0 | 0 | 13 | 0 | 13 |
| **S120** | 0 | 0 | 1 | 3 | 0 | 4 |
| **S121** | 0 | 0 | 0 | 2 | 0 | 2 |
| **S122** | 0 | 0 | 2 | 0 | 0 | 2 |
| **S123** | 0 | 0 | 1 | 0 | 0 | 1 |
| **S124** | 0 | 1 | 0 | 0 | 0 | 1 |
| **S126** | 0 | 0 | 3 | 0 | 4 | 7 |
| **S127** | 0 | 0 | 0 | 1 | 0 | 1 |
| **S128** | 5 | 0 | 0 | 1 | 0 | 6 |
| **S129** | 0 | 0 | 0 | 1 | 0 | 1 |
| **S13** | 0 | 1 | 0 | 0 | 0 | 1 |
| **S130** | 0 | 3 | 0 | 2 | 0 | 5 |
| **S14** | 3 | 1 | 3 | 1 | 1 | 9 |
| **S145** | 1 | 0 | 0 | 0 | 0 | 1 |
| **S146** | 1 | 0 | 0 | 0 | 0 | 1 |
| **S147** | 1 | 0 | 0 | 0 | 0 | 1 |
| **S148** | 1 | 0 | 0 | 0 | 0 | 1 |
| **S149** | 1 | 0 | 0 | 0 | 0 | 1 |
| **S150** | 0 | 0 | 0 | 0 | 3 | 3 |
| **S152** | 0 | 1 | 0 | 0 | 0 | 1 |
| **S153** | 0 | 1 | 0 | 0 | 0 | 1 |
| **S155** | 0 | 1 | 0 | 0 | 0 | 1 |
| **S156** | 0 | 0 | 1 | 2 | 0 | 3 |
| **S157** | 0 | 0 | 0 | 2 | 1 | 3 |
| **S158** | 0 | 0 | 0 | 3 | 0 | 3 |
| **S159** | 0 | 0 | 0 | 1 | 0 | 1 |
| **S160** | 0 | 0 | 0 | 1 | 0 | 1 |
| **S161** | 0 | 0 | 0 | 1 | 0 | 1 |
| **S162** | 0 | 1 | 0 | 0 | 0 | 1 |
| **S165** | 1 | 0 | 0 | 0 | 0 | 1 |
| **S166** | 0 | 0 | 0 | 3 | 0 | 3 |
| **S170** | 1 | 0 | 0 | 0 | 0 | 1 |
| **S171** | 0 | 0 | 0 | 1 | 0 | 1 |
| **S172** | 0 | 0 | 0 | 0 | 1 | 1 |
| **S173** | 0 | 0 | 0 | 0 | 1 | 1 |
| **S174** | 0 | 0 | 0 | 0 | 3 | 3 |
| **S176** | 0 | 0 | 0 | 0 | 1 | 1 |
| **S177** | 0 | 0 | 0 | 0 | 2 | 2 |
| **S178** | 0 | 0 | 0 | 0 | 2 | 2 |
| **S182** | 0 | 0 | 0 | 0 | 1 | 1 |
| **S27** | 0 | 2 | 2 | 0 | 0 | 4 |
| **S4** | 5 | 4 | 0 | 1 | 10 | 20 |
| **S5** | 1 | 1 | 0 | 4 | 0 | 6 |
| **S54** | 4 | 5 | 1 | 3 | 12 | 25 |
| **S55** | 0 | 3 | 13 | 3 | 4 | 23 |
| **S6** | 1 | 0 | 0 | 0 | 0 | 1 |
| **S63** | 0 | 0 | 0 | 0 | 1 | 1 |
| **S64** | 4 | 4 | 7 | 3 | 4 | 22 |
| **S66** | 3 | 6 | 3 | 3 | 3 | 18 |
| **S7** | 6 | 1 | 2 | 6 | 6 | 21 |
| **S74** | 0 | 3 | 1 | 3 | 2 | 9 |
| **S76** | 1 | 0 | 0 | 0 | 0 | 1 |
| **S78** | 1 | 2 | 7 | 2 | 0 | 12 |
| **S8** | 1 | 0 | 0 | 0 | 0 | 1 |
| **S82** | 0 | 2 | 0 | 0 | 0 | 2 |
| **S86** | 0 | 0 | 0 | 2 | 5 | 7 |
| **S90** | 1 | 0 | 0 | 0 | 0 | 1 |
| **S96** | 1 | 0 | 1 | 0 | 0 | 2 |
| **Total** | **67** | **60** | **81** | **74** | **81** | **363** |
